# Anthricin Isolated from *Anthriscus sylvestris* (L.) Hoffm. Inhibits the Growth of Breast Cancer Cells by Inhibiting Akt/mTOR Signaling, and Its Apoptotic Effects Are Enhanced by Autophagy Inhibition

**DOI:** 10.1155/2013/385219

**Published:** 2013-05-29

**Authors:** Chang Hwa Jung, Heemun Kim, Jiyun Ahn, Sung Keun Jung, Min Young Um, Kun-Ho Son, Tae Wan Kim, Tae Youl Ha

**Affiliations:** ^1^Department of Food Biotechnology, University of Science and Technology, Daejon 305-806, Republic of Korea; ^2^Division of Metabolism and Functionality Research, Korea Food Research Institute, 1201 Anyangpangyo-ro, Seongnam 463-746, Republic of Korea; ^3^Department of Food Science and Nutrition, Andong National University, Kyungbuk 760-749, Republic of Korea; ^4^Department of Food Science and Biotechnology, Andong National University, Kyungbuk 760-749, Republic of Korea

## Abstract

Anthricin (deoxypodophyllotoxin) is a natural product isolated from *Anthriscus sylvestris* (L.) Hoffm. (Apiaceae). Here, we investigated the effect of anthricin on autophagy and mammalian target of rapamycin (mTOR) signaling as anticancer actions in breast cancer cells. Many studies have supported the contention that the phosphoinositide 3-kinase (PI3K)/Akt/mTORC1 pathway is considerably deregulated in breast cancer and that autophagy plays important roles in the development of this type of cancer, although the exact underlying mechanisms remain unknown. Our data confirmed that anthricin markedly induced apoptosis in 2 breast cancer cell lines, MCF7 (estrogen receptor positive) and MDA-MB-231 (estrogen receptor, progesterone receptor, and Her2/Neu receptor negative). Anthricin treatment decreased the levels of phosphorylated Akt and mTORC1, followed by inhibition of cell growth. Interestingly, blockage of autophagy by a pharmacological inhibitor or genetic deletion of ULK1 and Atg13 accelerated anthricin-induced apoptosis, suggesting that autophagy has cytoprotective effects. Taken together, our results indicate that anthricin is an inhibitor of mTOR and that a combination of an autophagy inhibitor and anthricin may serve as a new promising strategy for the treatment of breast cancer cells.

## 1. Introduction

Breast cancer is the most common type of cancer in women, accounting for 22.9% of invasive cancers in this population and 16% of all female cancers [1]. Among the various therapeutic approaches that have been used to reduce the mortality from breast cancer, chemoprevention may be the most effective in reducing the risk of, or eradicating, cancer in healthy people or in patients with earlystage invasive breast cancer. Numerous studies have supported the contention that natural compounds can function as cancer-prevention and therapeutic agents. Several studies on breast cancer have revealed the potential of naturally occurring chemopreventive agents, such as eupatorin, oleuropein, genistein, and resveratrol, as anti-breast cancer drugs [2–6]. Chemoprevention is attractive for cancer therapy because it represents an easy and low-cost cancer-control method, mainly for individuals with an inherited predisposition to certain cancers.

*Anthriscus sylvestris* (L.) Hoffm. (Apiaceae) is a common wild plant that is indigenous to Europe, North America, Africa, Asia, and New Zealand [7]. The dried root of *A. sylvestris* has been used in Korean traditional drugs for the treatment of various diseases, including bronchitis, and as an antipyretic, a cough remedy, and an analgesic herbal drug. This plant accumulates the anthricin (deoxypodophyllotoxin), which has anticancer activity against lung cancer, lymphomas, and genital tumors [8–12]. The inhibitory effect of anthricin on a variety of cancer cells is related to the induction of G2/M cell-cycle arrest and caspase-dependent apoptosis [11]; however, the mechanism underlying this biological phenomenon remains unknown. Here, we sought to determine if the Akt/mammalian target of rapamycin (mTOR) pathway and the autophagic process play any specific role in the regulation of the anticancer properties of anthricin in breast cancer cell lines.

The Akt/mTOR pathway has been identified as an important target in breast cancer research over the past 20 years. This pathway is integral to various cellular functions, including cellular metabolism, proliferation, and survival [13, 14]. More specifically, this pathway overcomes drug resistance in hormone-receptor-positive breast cancer [13]. Although autophagy, which is a lysosome-mediated degradation system, may be important in the regulation of cancer development and progression and in determining the response of tumor cells to anticancer therapy, its role in cancer therapy remains controversial [15]. Autophagy is controlled by Akt/mTOR signaling in the regulation of nutrient-sensing pathways. mTOR represses the ULK1/2 complex, which recruits other autophagy-related proteins (Atg) for the formation of the autophagosome [16]. A recent report has suggested that autophagy acts as a prosurvival process that regulates apoptosis in breast cancer cells [17]. Therefore, we hypothesized that anthricin regulates Akt/mTOR signaling and autophagy to modulate cell death or cell survival. In this study, we evaluated the mechanism of cell death induced by anthricin isolated from *A. sylvestris *in MCF-7 and MDA-MB-231 breast cancer cell lines.

## 2. Materials and Methods

### 2.1. Isolation of Anthricin from *A. sylvestris* (L.) Hoffm

The dried roots of *A. sylvestris* (8.25 kg) were refluxed with hot MeOH (3 times) and concentrated to give a residue (959.25 g), which was suspended in H_2_O and was partitioned with hexane (89.41 g), CH_2_Cl_2_ (28.66 g), EtOAc (8.15 g), and BuOH (42.58 g). The hexane fraction (73.62 g) was loaded onto a silica-gel column (80 × 15 cm) and eluted with a gradient of hexane : EtOAc (10 : 0.2 to 10 : 1) to give 16 subfractions. Among these, subfraction 14 was recrystallized from MeOH to afford anthricin (2.9 g). The CH_2_Cl_2_ fraction (27.57 g) was loaded onto a silica-gel column (80 × 10 cm) and eluted with a gradient of hexane : EtOAc (10 : 1 to 10 : 2) to give 14 subfractions. Subfraction 12 was recrystallized from MeOH to afford anthricin (5.4 g). The molecular weight and fragment ions of the compound were identified using liquid chromatography-tandem mass spectrometry (LC-MS/MS) and nuclear magnetic resonance (NMR). The molecular weight and the fragment ions are summarized as follows: anthricin, C_22_H_22_O_7_; mp, 168–170°C; ^1^H-NMR (300 MHz, CDCl_3_): *δ* 6.65 (1H, s, H-2), 6.50 (1H, s, H-5), 6.32 (2H, s, H-2′, 6′), 5.93 (1H, d, *J* = 1.2 Hz, OCH_2_O), 5.91 (1H, d, *J* = 1.2 Hz, OCH_2_O), 4.58 (1H, d, *J* = 2.7 Hz, H-7′), 4.43 (1H, m, H-9), 3.90 (1H, m, H-9), 3.78 (3H, s, C_4′_-OCH_3_), 3.73 (6H, s, C_3′_-OCH_3_, C_5′_-OCH_3_), 3.05 (1H, m, H-7), 2.75 (2H, m, H-8, 8′); ^13^C-NMR (75.5 MHz, CDCl_3_) *δ*: 174.9 (C-9′), 152.5 (C-3′), 147.0 (C-3), 146.7 (C-4), 137.0 (C-4′), 136.3 (C-1′), 130.6 (C-6), 128.3 (C-1), 110.4 (C-5), 108.4 (C-6′), 108.2 (C-2), 101.2 (OCH_2_O), 72.0 (C-9), 60.7 (C_4′_-OCH_3_), 56.2 (C_3′_-OCH_3_, C_5′_-OCH_3_), 47.5 (C-8′), 43.7 (C-7′), 33.1 (C-7), 32.7 (C-8); EI-MS m/z: 398 [M]^+^.

### 2.2. Reagents

RPMI-1640 medium, fetal bovine serum (FBS), bovine calf serum (CS), sodium pyruvate, and penicillin-streptomycin were obtained from Gibco BRL (Grand Island, NY, USA). Anti-p62 (sc-28359), anti-mTOR (sc-1549), anti-ULK1 (sc-33182), anti-*β*-actin (sc-47778), anti-raptor (sc-81537), TSC2 (sc-893), and secondary antibodies were purchased from Santa Cruz Biotechnology (Santa Cruz, CA, USA). Antibodies against p-mTOR (no. 5536s), mTOR (no. 9272), p-S6K1 (no. 9205), S6K1 (no. 9202), p-Akt (no. 9271), Akt (no. 9272), LC3B (no. 2775s), PARP (no. 9542s), cleaved caspase-3 (no. 9661s), cleaved caspase-7 (no. 9491), cleaved caspase-9 (no. 9501), cyclin B1 (no. 4138), p-chk2 (no. 2661s), and Bax (no. 2772) were from Cell Signaling (Danvers, MA, USA). The anti-Atg13 antibody has been described in our previous report [16]. Glutathione 4B beads were obtained from GE Healthcare (Piscataway, NJ, USA). Chloroquine (CQ) and rapamycin were purchased from Sigma-Aldrich (Saint Louis, MO, USA) and Calbiochem (San Diego, CA, USA), respectively.

### 2.3. Cell Culture and Transfection

MCF7 and MDA-MB-231 cells were cultured in RPMI with 1% penicillin-streptomycin and 10% FBS at 37°C in 5% CO_2_. For transient transfection, cells were transfected with GFP-LC3 using FuGENE 6 (Roche Applied Science, Indianapolis, IN) according to the manufacturer's protocol.

### 2.4. RNA Interference

RNA silencing was achieved using the ON-TARGETplus SMART human TSC2 pool or ON-TARGETplus siCONTROL nontargeting pool (Dharmacon, Lafayette, CO, USA). MCF-7 cells were seeded in 60-mm plates and transiently transfected with negative control siRNA or TSC2 siRNA using Lipofectamine RNAiMAX (Invitrogen) according to the manufacturer's instructions. Cells were treated with 25 *µ*g/mL anthricin for 12 h at 24 h after transfection and analyzed by Western blotting.

### 2.5. Lentiviral Preparation and Viral Infection

Lentiviral shRNA transduction was assessed as described previously [16]. Briefly, the PLKO.1 vectors encoding shRNAs were transfected into HEK 293T cells with the lentiviral packaging vectors pHR8.2ΔR and pCMV-VSV-G using FuGENE 6. Viruses were collected 72 h after transfection, and MCF-7 cells were infected with the collected viruses for 8 h in the presence of polybrene. Stably transduced cells were selected using puromycin. The target sequences for the Atg13 shRNA were 5′-gaatttggagctggaggat-3′ and 5′-agtttcctacacggtgtac-3′, and those for the ULK1 shRNA were 5′-gacttccaggaaatggctaat-3′ and 5′-acatcgagaacgtcaccaagt-3′. Knockdown of *Atg13* and *ULK1* was confirmed by immunoblotting.

### 2.6. Cell Viability Assay

The human breast cancer cell lines were seeded in a 96-well plate at a concentration of 2 × 10^3^ cells/well. After 24 h of preconditioning, the cells were exposed to various concentrations of anthricin for 12 or 24 h, respectively. Subsequently, 50 *μ*L of cell counting kit-8 (no. CK04, Dojindo, Kumamoto, Japan) solution was added into each well and the plate was incubated for an additional 3 h at 37°C, to detect cell survival. Cell viability was calculated by measuring the absorbance on a microplate reader (Tecan, Infinite M200) at 450 nm.

### 2.7. Cell Proliferation Assay

The breast cancer cell lines were treated with anthricin and cells were counted on days 1, 2, and 3. On each of these days, cells were trypsinized and harvested with RPMI medium. Cells were diluted 10 times with Isoton II diluent and loaded onto a Z2 counter. Three independent measurements were analyzed quantitatively.

### 2.8. Western Blot Assay

The breast cancer cell lines were treated with anthricin for the indicated times. After treatment, cells were harvested with 1% Triton-X100 buffer, run on SDS-PAGE, transferred to a PVDF membrane, and probed with polyclonal or monoclonal antibodies.

### 2.9. Coimmunoprecipitation

For coimmunoprecipitation, whole-cell lysates were prepared in a buffer containing 0.3% Chaps buffer, as described by Kim et al. [18], and immunoprecipitated with an mTOR antibody. Immunoprecipitated proteins were washed 4 times using lysis buffer, loaded onto 8% gels, transferred onto a PVDF membrane, and detected.

### 2.10. Immunostaining

MCF7 cells were seeded and transfected with GFP-LC3 on a Lab-TEK Chamber Slide (no. 177437, Thermo Fisher Scientific, Rochester, NY). Cells were treated with anthricin for 8 h at 2 d after transfection and fixed with 3.7% formaldehyde, permeabilized with 0.3% Triton X-100, and stained with DAPI (4′-6-diamidino-2-phenylindole; D-1306, Invitrogen). Images from stained cells were acquired using a confocal microscope (Nikon, DIGITAL ECLIPSE C1 plus, Japan).

### 2.11. Annexin V Apoptosis Analyses

Cells (1 × 10^3^) were plated in 60 mm plates and treated with vehicle, anthricin, or chloroquine for 12 h. Cells were then fixed in 70% ethanol and stored at −20°C for 24 h. After staining with annexin V, apoptosis was determined using a BD FACS Calibur Flow Cytometer (BD Biosciences, San Jose, CA).

### 2.12. Statistical Analyses

Differences between groups were evaluated using one-way analysis of variance (ANOVA) with the GraphPad Prism5 software (San Diego, CA, USA). The Bonferroni post hoc test was used if differences were significant after ANOVA. Data are expressed as the mean ± SD.

## 3. Results

### 3.1. Anthricin from *A. sylvestris* (L.) Hoffm. Induces Apoptosis and Cell-Cycle Arrest in Breast Cancer Cells

**Figure 1:**
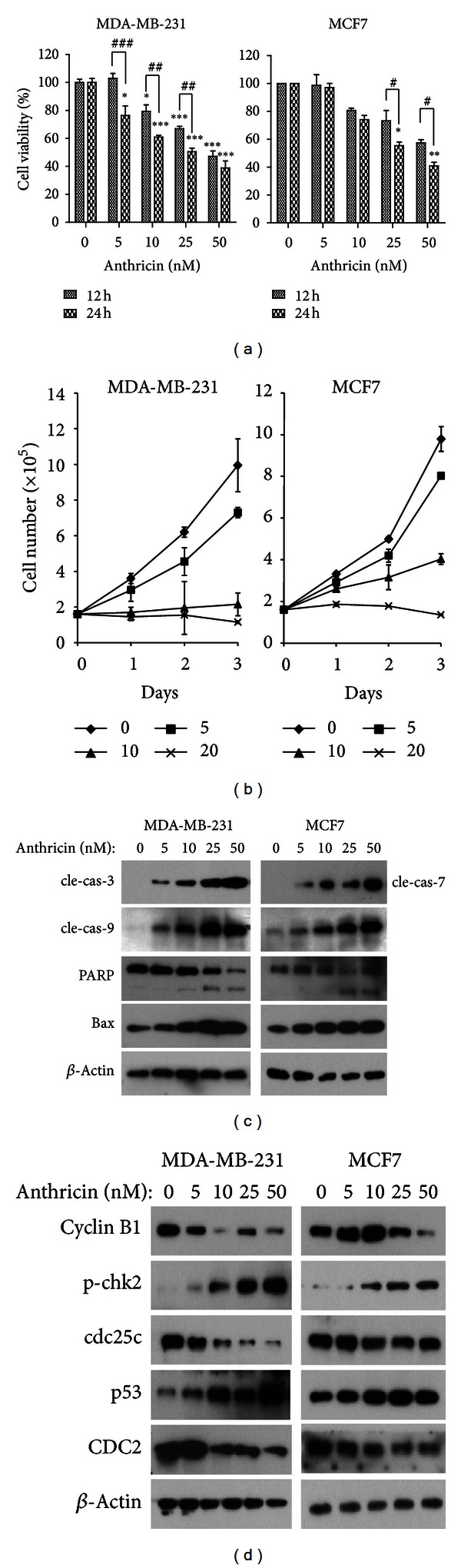
Anthricin isolated from *Anthriscus sylvestris* (L.) Hoffm. inhibits cell growth and proliferation. (a) Analysis of cell viability in anthricin-treated MDA-MB-231 and MCF7 cells. Cells were treated with varying concentrations of anthricin for 12 or 24 h. Cell viability was evaluated using the cell counting kit-8 (CCK-8). Data are expressed as a percentage of the control and shown as mean ± SD (*n* = 3) values. _ _*P < 0.05; _ _**P < 0.01; _ _***P < 0.001 compared with vehicle-treated cells. _ _^#^ *P* < 0.05; _ _^##^ *P* < 0.01; _ _^###^ *P* < 0.001 compared with the group treated with anthricin for 12 h. (b) Reduced cell proliferation rate of breast cancer cells. The error bars represent the mean ± SD (*n* = 4). (c) Induction of apoptosis by anthricin in breast cancer cells. The extent of apoptosis in MDA-MB-231 and MCF-7 cells treated with varying concentrations of anthricin for 24 h was analyzed by immunoblotting. (d) Cell cycle arrest by anthricin in breast cancer cells. The expression of cell-cycle-related proteins was analyzed by immunoblotting.

To investigate the inhibitory effect of anthricin on breast cancer cell growth, we first determined cell viability in 2 breast cancer cell lines: MCF7 (estrogen receptor positive) and MDA-MB-231 (estrogen receptor, progesterone receptor, and Her2/Neu receptor negative). The cells were treated with various concentrations of anthricin for the indicated time, and cell viability was analyzed using a cell counting kit-8. Anthricin significantly inhibited cell growth and proliferation in both cancer cell lines in a dose-dependent manner without toxicity in normal cells (Figures 1(a) and 1(b), Supplementary 1 available online at http://dx.doi.org/10.1155/2013/385219). The IC50 concentrations at 24 h in anthricin-treated MDA-MB-231 and MCF7 cells were 40.9 ± 2.1 and 41.1 ± 1.5 nM, respectively. A previous study showed that anthricin induces apoptosis in HeLa cells, suggesting the possible involvement of the induction of apoptosis in different cancer cell lines [11, 12]. To confirm the effect of anthricin on the induction of apoptosis, the cells were treated with anthricin and apoptosis-related signals were analyzed by immunoblotting. Anthricin treatment in both cancer cell lines promoted the time-dependent cleavage of caspase-3, caspase-7, or caspase-9 (Figure 1(c)). We also observed that PARP cleavage was augmented by increasing the concentration of anthricin. The drug also induced Bax accumulation in a dose-dependent manner. Previous studies showed that anthricin induces cell cycle arrest at G(2)/M phase in HeLa cells [11, 12]. To elucidate the mechanism of G2/M phase arrest in anthricin-treated breast cancer cells, we investigated the expression of G2/M phase-related factor. Our results confirmed that anthricin induced G(2)/M phase cell-cycle arrest in breast cancer cells by enhancing the expression of p53 and the phosphorylation of chk2 and downregulating Cyclin B1, cdc25c, and CDC2 (Figure 1(d)). G2/M phase arrest is linked in the activation of ATM, chk2, p53, and p21 as well as the inactivation of CDC2 [19]. Collectively, these data suggest that anthricin treatment exhibited potent growth suppressive activity in breast cancer through induction of apoptosis and G(2)/M phase cell-cycle arrest.

### 3.2. Anthricin Induces Autophagy

**Figure 2:**
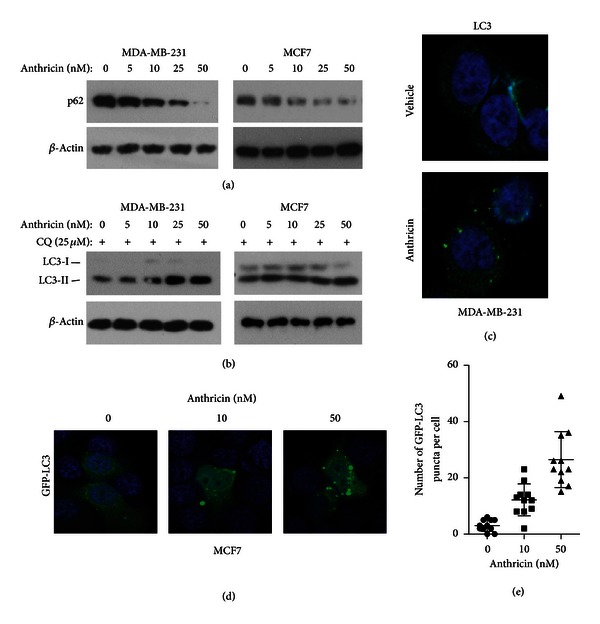
Anthricin induces autophagy. (a) p62 levels in anthricin-treated breast cancer cells. Cells were treated with varying concentrations of anthricin for 8 h. Protein levels were analyzed by immunoblotting. (b) Immunoblot analysis of the LC3 protein. The levels of endogenous LC3-II increased in breast cancer cells treated with anthricin. The cells were cotreated with anthricin and chloroquine (CQ) for 8 h. (c) Immunostaining of LC3 in MDA-MB-231 cells. The cells were treated with CQ for 8 h to induce autophagosome accumulation. After fixation and permeabilization, autophagosomes were stained with an anti-LC3B rabbit polyclonal antibody and visualized with Alexa Fluor 488 goat anti-rabbit IgG. (d) MCF-7 cells were transduced with GFP-LC3. Two days later, cells were cotreated with anthricin and CQ for 8 h. (e) Quantitative analysis of the formation of GFP-LC3 puncta.

Autophagy is a lysosomal degradation pathway for quality control of cytoplasm by eliminating dysfunctional subcellular structures [20]. Recently, autophagy has been suggested to be responsible for the maintenance of intracellular homeostasis and for enabling cell survival under stress conditions. This process is involved in the pathogenesis of various diseases, including cancer [15]. Recent studies on autophagy seem to be controversial regarding its dual role as a mechanism that is responsible for protecting or killing cells [21]. We examined whether anthricin induces autophagy and whether autophagy subsequently promotes or prevents apoptosis in anthricin-treated cells. We first evaluated the level of p62, a ubiquitin-binding protein that is involved in autophagy and the levels of which are decreased by lysosomal hydrolases during autophagic process [22]. Anthricin treatment downregulated p62 in MDA-MB-231 and MCF7 cells in a dose-dependent manner (Figure 2(a)). We confirmed this result by evaluating the lipidation of microtubule-associated protein 1 light chain 3 (LC3) via immunoblotting. During autophagy, cytosolic LC3-I conjugates covalently to phosphatidylethanolamine to yield a lipidated form of LC3 (LC3-II) [23]. The combination with the lysosomal inhibitors, CQ, is increased in LC3-II levels due to the inhibition of LC3-II degradation by lysosomes [23]. CQ inhibits late step of autophagy pathway as it raises the lysosomal pH, which leads to inhibition of both fusion of autophagosome with lysosome [24]. This indicates that an increase in the total amount of LC3-II in the presence of lysosomal inhibitor indicates an increase of autophagic influx. Anthricin treatment gradually increased the LC3-II form in a dose-dependent manner in the presence of CQ (Figure 2(b)), suggesting that anthricin plays a role in inducing autophagy. We also investigated the formation of endogenous LC3 puncta in MDA-MB-231 cells using fluorescence microscopy. Our confocal images showed that LC3 puncta formation was abundant in cells treated for 8 h (Figure 2(c)). We obtained similar results in MCF-7 cells, as anthricin treatment increased the formation of LC3 puncta in MCF-7 cells transiently transfected with GFP-LC3 (Figures 2(d) and 2(e)).

### 3.3. Autophagy Plays a Cytoprotective Role in Anthricin-Treated Breast Cancer Cells

**Figure 3:**
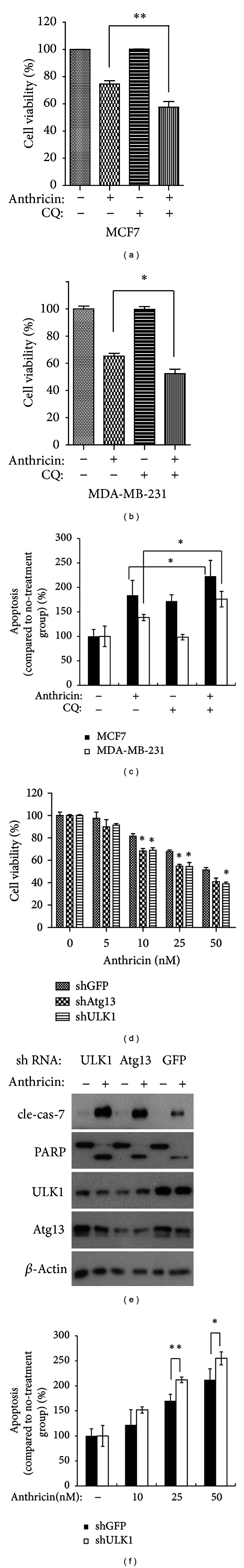
Autophagy plays a cytoprotective role in anthricin-treated breast cancer cells. (a and b) Autophagy inhibition promotes cell death. Cells were treated with 25 nM anthricin and/or 25 *µ*M CQ for 12 h. Data are expressed as a percentage of the control and shown as mean ± SD (*n* = 3). _ _*P < 0.05; _ _**P < 0.01 compared to vehicle-treated cells. (c) Cotreatment of anthricin and CQ is more sensitive to the apoptotic cell death in MCF-7 and MDA-MB-231. Apoptosis was analyzed by flow cytometry and annexin V staining as described in Materials and Methods. Data are represented as means ± S.D. as determined from 3 independent experiments. _ _*P < 0.05 compared to anthricin alone. (d) Anthricin enhances cell death in ULK1 or Atg13 knockdown cells. MCF-7 cells stably transduced with lentiviral shRNA were treated with anthricin (25 nM) for 12 h. Data are expressed as a percentage of the control and shown as the mean ± SD (*n* = 3). _ _*P < 0.05; _ _**P < 0.01 compared with shGFP control cells within the same concentration. (e) Apoptosis increased in ULK1- and Atg13-silenced MCF-7 cells after anthricin treatment. The extent of apoptosis in cells treated with 25 nM anthricin for 12 h was analyzed by immunoblotting. (f) Anthricin is more sensitive to the apoptotic cell death in ULK1-silenced MCF-7 cells. Apoptosis was analyzed by flow cytometry and annexin V staining. _ _*P < 0.05 compared with shGFP control cells within the same concentration.

Although the results described earlier suggest that anthricin induces autophagy, it remains to be determined whether the inhibition of autophagy promotes or prevents apoptosis after breast cancer cells are treated with anthricin. To address this question, we blocked autophagy via pharmacological or genetic inhibition. The blockage of autophagy using CQ enhanced cell death in MCF7 and MDA-MB-231 cells without any change in CQ-induced cell death (Figures 3(a) and 3(b), Supplementary 2). We also confirmed that cotreatment with CQ enhanced apoptosis as detected by annexin V staining (Figure 3(c)). However, apoptotic effect was not enhanced by cotreatment with rapamycin (Supplementary 3). These results suggest that mTOR activity may directly affect the cell growth in breast cancer cells. To validate the finding that the inhibition of autophagy promotes cell death in MCF-7 cells, the autophagy-related proteins ULK1 and Atg13 were silenced in MCF-7 cells using lentiviral infection. The Atg13- and ULK1-silenced cells exhibited a significant increase in cell death after exposure to anthricin (Figure 3(d)). Western blotting also indicated that the expression levels of cleaved caspase-7 and PARP cleavage increased in ULK1 and Atg13 knockdown cells compared with wild-type cells (Figure 3(e)). Atg13 plays a positive role in the regulation of ULK1 activity and is important for the stability of ULK1 [16]. Figure 3(e) also supports that Atg13 is important for ULK1 stabilization in MCF7 cells. Collectively, these results suggest that blockage of autophagy enhances anthricin-induced apoptosis in breast cancer cells.

### 3.4. Anthricin Inhibits Akt-mTOR Signaling

**Figure 4:**
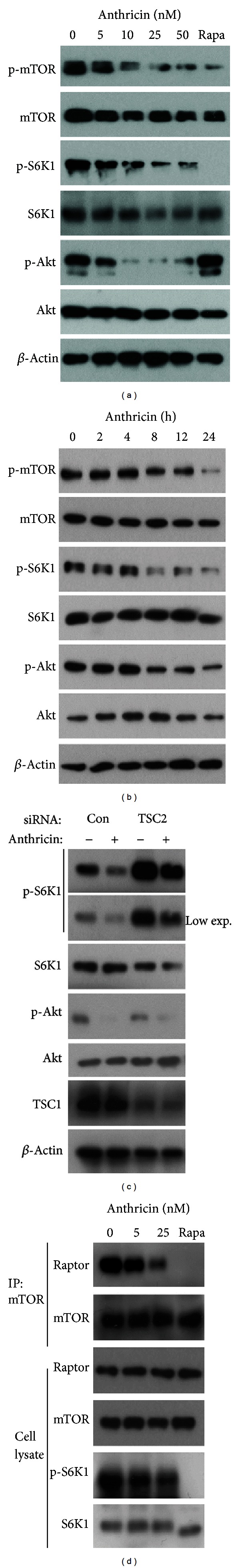
Anthricin inhibits Akt/mTOR signaling. (a) Anthricin inhibits the phosphorylation of mTOR, S6K1, and Akt in a dose-dependent manner. MCF-7 cells were treated with varying concentrations of the drug for 12 h or 100 nM rapamycin for 1 h, and the phosphorylation states of mTOR (p-Ser2448), S6K1 (p-Thr389), and Akt (p-Ser473) were analyzed by immunoblotting. (b) Anthricin inhibits the phosphorylation of mTOR, S6K1, and Akt in a time-dependent manner. MCF-7 cells were treated with 25 nM anthricin for the time period specified. (c) Anthricin is a potential target of mTOR and Akt. MCF-7 cells were transduced with TSC2 siRNA or scrambled siRNA (control). Cells were treated with or without 25 nM anthricin, and the proteins were analyzed by immunoblotting. (d) Anthricin disrupts the association between mTOR and raptor. mTOR immunoprecipitates were isolated from anthricin-treated MCF-7 cells by using an anti-mTOR antibody, and the amounts of raptor and mTOR in the immune complexes were analyzed by immunoblotting.

The mTOR pathway promotes tumor growth and survival, while suppressing autophagy [25, 26]. Anthricin treatment strongly inhibited mTOR kinase activity in a time- and dose-dependent manner, as measured by the phosphorylation of S6K1 (Figures 4(a) and 4(b)). The reduced mTOR kinase activity seems to be due to the inhibition of Akt. The siRNA knockdown of TSC2 in MCF-7 cells affected mTOR activity slightly after anthricin treatment, suggesting that anthricin may directly inhibit both Akt and mTOR signals (Figure 4(c)). Raptor is an mTOR-binding protein; its deletion or the disruption of its binding to mTOR appears to increase apoptosis [27]. We also observed that anthricin disrupted the association between raptor and mTOR. These results indicate that anthricin treatment to breast cancer cells induces apoptosis via the inhibition of Akt-mTOR signals, whereas autophagy induction may act as a cell-survival mechanism in MCF-7 cells.

## 4. Discussion

*Anthriscus sylvestris* (L.) Hoffm. has been used in traditional medicine for the treatment of various diseases, such as bronchitis, as well as an antipyretic and analgesic herbal drug [7]. *A. sylvestris* contains lignans such as anthricin (deoxypodophyllotoxin) that are responsible for its various biological effects. In this study, we isolated anthricin from *A. sylvestris* and evaluated the mechanism underlying the apoptosis induced by treatment with this compound in breast cancer cells. We found that mTOR signaling and autophagy play important roles in the balance between cell death and cell survival induced by anthricin in breast cancer cells.

Several groups have reported that anthricin has antitumor activity against prostate, cervical, and lung cancer cells [10, 11, 28]. Our data showed that anthricin was effective in inducing apoptosis in the MCF-7 and MDA-MB-231 breast cancer cell lines. Anthricin treatment induced autophagy, which has a dual function in cancer cell lines (death and survival). Recent studies have suggested that autophagy plays a cytoprotective role in breast cancer cells [29, 30]. Our data for autophagy flux assays and immunostaining demonstrated that anthricin induced autophagy. An increase in autophagosome accumulation may be caused either by increased autophagosome formation or by the blockage of autophagic degradation after fusion of the autophagosome with a lysosome. In the autophagy flux assay, we observed a decrease in the levels of p62, which is degraded in the autolysosome. The formation of endogenous LC3 or GFP-LC3 puncta was increased in anthricin-treated breast cancer cells. Collectively, these data demonstrate that anthricin is a positive regulator of the autophagic process.

Our study provided strong evidence that anthricin induces apoptosis and autophagy. According to our results, autophagy plays an important role in the survival of breast cancer cells. Notably, apoptosis increased on cotreatment with anthricin and a pharmacological autophagy inhibitor, CQ. We also investigated whether the genetic knockdown of autophagy-related proteins enhanced apoptosis in MCF-7 cells. MCF-7 cells were silenced using shULK1 and shAtg13; these proteins play a critical role in the autophagic process [16]. The knockdown of ULK1 and Atg13 enhanced cell death in MCF-7 cells after treatment with anthricin. These results suggest that autophagy inhibition can be an apoptosis-enhancing pathway in breast cancer cells. Cells have developed a defense mechanism in response to changes in the intracellular environment. During stress, prosurvival and prodeath processes are concomitantly activated in cells. The final outcome depends on the balance between life and death during stress. If exposure to stress results in damage to organelles, then the cell can clear the damaged organelles via autophagy, but if the cell is beyond rescue, apoptosis will become.

Accumulating data suggest that mTOR is an attractive target for the development of novel anticancer molecules. More specifically, the phosphatidylinositol-3-kinase (PI3K)/Akt/mTOR pathway plays a critical role in multiple cellular functions, including proliferation, growth, and metabolism, and this pathway is highly activated in many types of cancer [13, 25]. Our data showed that anthricin treatment reduced the phosphorylation of Akt, S6K1, and mTOR, suggesting that anthricin inhibits the Akt/mTOR signaling pathway, thus inhibiting breast cancer growth. In fact, this pathway can modulate estrogen-independent growth, which may lead to endocrine resistance [31]. Anthricin also induced autophagy, which is directly regulated by mTOR. Autophagy induction under unfavorable conditions may play a cytoprotective role in breast cancer cells. Recent data suggest that coordinate inhibition of the mTOR and autophagy pathways promotes apoptosis [32], and these findings may require further preclinical and clinical study of coordinate autophagy and Akt/mTOR inhibition as a rational approach to improve therapeutic outcomes in breast cancer is warranted.

In conclusion, our most significant finding is that anthricin isolated from *A. sylvestris* inhibits Akt/mTOR signaling in breast cancer cells. Thus, anthricin inhibits the growth of breast cancer cells and its apoptotic effects are enhanced by autophagy inhibition. The current study shows that anthricin may be an effective as an anti-breast cancer agent. Our data predict that both autophagy and mTOR inhibition may be useful therapeutic approaches for breast cancer.

## Supplementary Material

The cells were treated with varying concentrations of anthricin for 12h. Cells were stained with annexin *V and were determined using a FACS (Supplementary 1). To investigate whether autophagy blockage enhances cell death, cells were treated with 25 nM anthricin and/or increasing concentrations of CQ. Cell viability and LC3II formation were analyzed using a cell counting kit-8 and immunoblotting, respectively (Supplementary 2(a) and 2(c))*. MDA-MB-231 cells were also treated with anthricin and/or rapamycin for 12h, and the expression of p-S6K1 and p-Akt in all groups were assayed by immunoblotting and the cell viability were analyzed by *a cell counting kit-8* (Supplementary 3(a) and 3(b)).
