# Supplementary figures and images for: Anthricin Isolated from Anthriscus sylvestris (L.) Hoffm. Inhibits the Growth of Breast Cancer Cells by Inhibiting Akt/mTOR Signaling, and Its Apoptotic Effects Are Enhanced by Autophagy Inhibition
Source: Evid Based Complement Alternat Med. 2013 May 29;2013:385219. doi: 10.1155/2013/385219 (PMC3681310; doi:10.1155/2013/385219)

(A)

|            |   |   |   |   |
|------------|---|---|---|---|
| Anthracin: | - | - | + | + |
| RM:        | - | + | - | + |

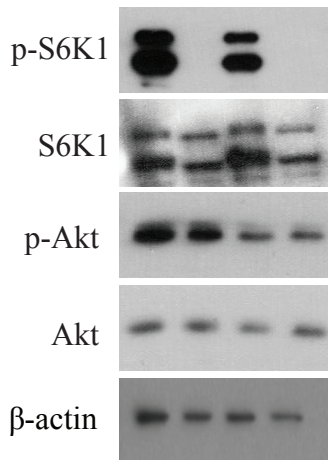

(B)

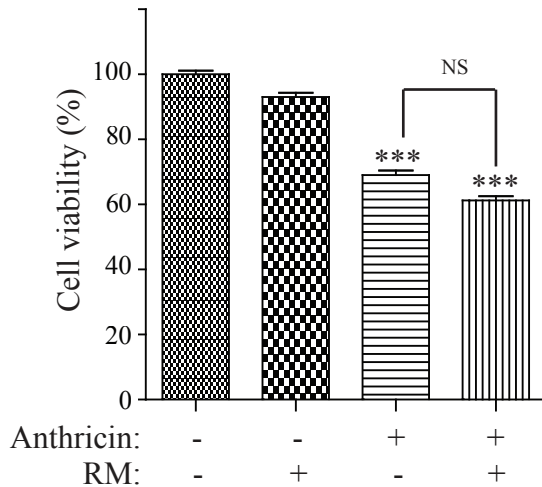

Supplement: Supplementary file 1 — The cells were treated with varying concentrations of anthricin for 12h. Cells were stained with annexin V and were determined using a FACS (Supplementary 1). To investigate whether autophagy blockage enhances cell death, cells were treated with 25 nM anthricin and/or increasing concentrations of CQ. Cell viability and LC3II formation were analyzed using a cell counting kit-8 and immunoblotting, respectively (Supplementary 2(a) and 2(c)). MDA-MB-231 cells were also treated with anthricin and/or rapamycin for 12h, and the expression of p-S6K1 and p-Akt in all groups were assayed by immunoblotting and the cell viability were analyzed by a cell counting kit-8 (Supplementary 3(a) and 3(b)). [file 385219.f1.pdf]
